# Genome-resolved analysis reveals disruption of gut microbial vitamin B and K_2_ biosynthesis during *Toxoplasma gondii* infection in mice

**DOI:** 10.1128/spectrum.01564-26

**Published:** 2026-09-08

**Authors:** Hai-Long Yu, Hany M. Elsheikha, Hai-Ping Wang, Ying-Qian Gao, Rui Liu, He Ma, Jing Jiang, Ying Li, Xiao-Xuan Zhang

**Affiliations:** 1 College of Life Sciences, Changchun Sci-Tech University 381875 , Changchun, Jilin, People's Republic of China; 2 College of Food and Pharmaceutical Engineering, Wuzhou University 215296, Wuzhou, Guangxi, People's Republic of China; 3 College of Veterinary Medicine, Jilin Agricultural University 85112, Changchun, Jilin, People's Republic of China; 4 Faculty of Medicine and Health Sciences, School of Veterinary Medicine and Science, University of Nottingham, Sutton Bonington Campus, Loughborough, United Kingdom; 5 College of Veterinary Medicine, Qingdao Agricultural University 98431, Qingdao, Shandong, People's Republic of China; Clemson University, Clemson, South Carolina, USA

**Keywords:** *Toxoplasma gondii*, gut microbiome, mouse, B vitamins, vitamin K_2_, metagenome-assembled genomes, niacin

## Abstract

*Toxoplasma gondii* infection remodels the gut microbiome, yet its impact on microbial vitamin biosynthetic potential and host redox metabolism remains unclear. Here, we integrated mouse gut metagenomes with publicly available metagenome-assembled genomes (MAGs) to construct a genome-resolved atlas of B-vitamin and vitamin K_2_ biosynthesis. From 45,697 MAGs, we curated 4,771 representative genomes, of which 2,682 met high-quality criteria (completeness ≥90%, contamination <5%). Functional annotation identified 229,717 vitamin-related genes corresponding to 177 Kyoto Encyclopedia of Genes and Genomes (KEGG) orthologs across *de novo* pathways for eight B vitamins, thiamine (B_1_), riboflavin (B_2_), niacin (B_3_), pantothenate (B_5_), pyridoxine (B_6_), biotin (B_7_), folate (B_9_), cobalamin (B_12_), and vitamin K_2_. Among the high-quality genomes, 1,665 encoded complete *de novo* pathways for at least one vitamin, highlighting functional specialization and community-level complementarity. Transcripts per million-normalized metagenomic read counts revealed significant differences in KEGG ortholog abundances across six of the nine vitamin pathways. Reanalysis of metagenomic data from infected mice (acute, chronic, and control; *n* = 10 per group) revealed a stage-dependent reduction in α-diversity of vitamin biosynthesis pathways during acute infection, and a clear β-diversity separation from chronic and control groups. Core niacin biosynthesis genes (*nadB*, *nadA*, *nadC*) displayed phylum-specific redistribution, indicating selective remodeling of microbial NAD^+^ precursor production under infection-induced metabolic stress. These results suggest that *T. gondii* infection disrupts cooperative vitamin biosynthetic networks while specifically modulating niacin pathways linked to host NAD^+^ metabolism.

**IMPORTANCE:**

Gut microbes can synthesize essential vitamins, but how infection alters this function is poorly understood. By integrating mouse gut metagenomes with genome-resolved microbial data, we show that *Toxoplasma gondii* infection reshapes the vitamin biosynthetic potential of the gut microbiome in a stage-dependent manner. Acute infection reduces the diversity of vitamin biosynthesis pathways and shifts the taxonomic distribution of key niacin biosynthesis genes involved in microbial NAD^+^ precursor production. These findings identify vitamin metabolism, especially niacin-related pathways, as a sensitive functional axis of microbiome remodeling during infection. Our work links microbial taxonomic changes to functional metabolic consequences and suggests that microbiome-mediated regulation of NAD^+^-related metabolism may contribute to host redox adaptation during *T. gondii* infection.

## INTRODUCTION

*Toxoplasma gondii* is an obligate intracellular parasite that infects a wide range of warm-blooded hosts and has a worldwide distribution (1, 2). Infection with *T. gondii* is associated with profound immunological and metabolic changes in the host (3, 4). Recent evidence indicates that these infection-associated alterations extend beyond host tissues and involve the gut microbiome (5). Previous studies have shown that *T. gondii* infection can induce gut microbial dysbiosis, alter the relative abundance of commensal bacterial lineages, reshape microbial community diversity, and perturb host-associated metabolic profiles (4). These findings highlight the role of infection-stage-dependent host–microbiome interactions in shaping metabolic responses over time. However, despite growing evidence that *T. gondii* infection alters gut microbial composition, it remains unclear whether these compositional changes are accompanied by alterations in the microbiome’s genome-encoded vitamin biosynthetic capacity and how such functional shifts may influence host metabolic adaptation across different stages of infection.

One of the key functional contributions of the gut microbiome is the *de novo* biosynthesis of vitamins, including eight B vitamins, thiamine (B_1_), riboflavin (B_2_), niacin (B_3_), pantothenate (B_5_), pyridoxine (B_6_), biotin (B_7_), folate (B_9_), and cobalamin (B_12_), as well as vitamin K_2_ (menaquinone) (6). These micronutrients were selected because they represent major microbiome-derived cofactors with broad relevance to both host and microbial physiology. B vitamins participate in central metabolic processes, including energy production, amino acid and fatty acid metabolism, redox reactions, nucleotide biosynthesis, and immune regulation (7, 8). Vitamin K_2_ is mainly produced by bacteria and plays an important role in microbial electron transport and anaerobic respiration; it has also been linked to host physiological processes, including bone and cardiovascular health (7, 9). Therefore, B-vitamin and vitamin K_2_ biosynthetic pathways provide a biologically meaningful functional readout for evaluating how infection may perturb microbiome-mediated micronutrient metabolism.

Microbial vitamin production is often highly specialized, and vitamin biosynthetic pathways are widely distributed across gut bacterial taxa. This creates a community-level network of metabolic flexibility and complementarity, in which distinct microbes contribute different biosynthetic steps (10, 11). Although *T. gondii*-associated gut microbiota alterations have been increasingly reported, whether these infection-driven taxonomic shifts translate into functional changes in microbial vitamin biosynthetic potential has not been systematically examined.

Among these vitamins, pathways linked to NAD^+^ metabolism may be particularly important during infection, as immune activation and metabolic reprogramming substantially increase cellular demand for NAD^+^. Niacin occupies a particularly critical position because it is the precursor of nicotinamide adenine dinucleotide (NAD^+^) and NADP^+^, essential redox cofactors that boost mitochondrial biogenesis and regulate cellular energy balance, oxidative stress responses, DNA repair, and immune signaling (12). NAD^+^ metabolism is tightly linked to immunometabolic regulation, and its availability influences inflammatory signaling pathways and cellular activation states (13). During infection and inflammation, immune cell activation further depletes NAD^+^ pools through metabolic reprogramming toward glycolysis and the activity of NAD^+^-consuming enzymes such as poly(ADP-ribose) polymerases and sirtuins (14). Microbial metabolism can contribute to host NAD^+^ pools through the production and transformation of NAD^+^ precursors, highlighting a potential link between gut microbiota and host redox homeostasis (15). Consequently, alterations in microbial niacin biosynthetic capacity during infection may have functional implications for host metabolic adaptation.

Understanding how *T. gondii* infection reshapes the microbiome’s vitamin biosynthetic capacity requires approaches that resolve metabolic functions at high taxonomic resolution. Metagenome-assembled genomes (MAGs) provide a powerful framework for characterizing microbiome function at the level of individual genomes (16). By combining high-quality MAGs with shotgun metagenomic profiling, it is possible to reconstruct complete biosynthetic pathways, quantify their abundance, and pinpoint the taxonomic contributors to specific metabolic functions (16–18). This genome-resolved approach enables detailed dissection of distributed pathways, such as vitamin biosynthesis, and facilitates the detection of infection-associated functional reprogramming within microbial communities (9).

In this study, we aimed to determine whether *T. gondii* infection alters the vitamin biosynthetic potential of the mouse gut microbiome in an infection-stage-dependent manner. The study consisted of two major analytical components. First, we constructed a non-redundant mouse gut genome catalog by integrating publicly available MAGs from the Comprehensive Mouse Microbiota Genome (CMMG) catalog (19) and MAGs reconstructed from *T. gondii*-associated metagenomic samples. This catalog was used to characterize the genomic potential and taxonomic distribution of *de novo* biosynthetic pathways for eight B vitamins and vitamin K_2_. Second, we reanalyzed shotgun metagenomic data from control mice and *T. gondii*-infected mice at acute and chronic infection stages to quantify infection-associated changes in vitamin biosynthesis-related Kyoto Encyclopedia of Genes and Genomes (KEGG) orthologs (KOs), pathway diversity, and taxonomic contributors. Particular attention was given to niacin biosynthesis because of its direct connection to NAD^+^ metabolism and host redox homeostasis. Through this genome-resolved framework, we sought to link infection-associated changes in microbial community structure with functional shifts in microbiome-derived micronutrient metabolism.

## MATERIALS AND METHODS

### Study design and data sets

This study consisted of two major analytical components. First, we constructed a genome-resolved mouse gut microbiome catalog to characterize the vitamin biosynthetic potential of mouse gut microorganisms. This catalog included 45,697 MAGs, comprising 41,798 MAGs from the CMMG catalog (https://ezmeta.unige.ch/CMMG/) (19) and 3,899 MAGs from the National Center for Biotechnology Information (NCBI; accession PRJNA1419542).

Second, we assessed infection-associated changes in microbial vitamin biosynthesis using 30 publicly available mouse gut shotgun metagenomic samples from NCBI BioProject PRJNA1419542, including acute *T. gondii* infection (AI), chronic *T. gondii* infection (CI), and uninfected control groups, with 10 samples per group. The original infection experiment was conducted using SPF female C57BL/6J mice aged 6–8 weeks. The mice were housed in a pathogen-free facility equipped with an independent ventilation system at 22 ± 2°C and 50% ± 10% relative humidity under a 12 h light/12 h dark cycle, with food and water provided *ad libitum*, and were acclimated for 1 week before the experiment. The mice were then randomly assigned to the control, AI, and CI groups. Mice in the AI and CI groups were orally inoculated with brain cysts of the type II PRU strain of *T. gondii*, whereas control mice received an equivalent volume of PBS.

### Data sets and bioinformatic analysis

Quality assessment of the 45,697 MAGs was performed using CheckM2 v.1.0.1 (20), evaluating genome completeness and contamination. MAGs were retained for downstream analyses if they met the following criteria: contamination <5%, completeness ≥50%, and a genome quality score (calculated as completeness − 5 × contamination) ≥50. After filtering, 40,646 MAGs were retained. Taxonomic annotation of the filtered MAGs was performed using the classify_wf workflow in GTDB-Tk v.2.3.2 (21), with the GTDB database as the reference. To reduce strain-level redundancy, genomes were dereplicated using dRep v.3.4.5 (22), retaining representative genomes with an average nucleotide identity (ANI) <99%. Dereplication was performed with the following parameters: -pa 0.9 -sa 0.99 -nc 0.30 -p 40 -cm larger --S_algorithm fastANI. This process resulted in 4,771 non-redundant genomes for subsequent analyses.

### Gene prediction and functional annotation

Open reading frames were predicted from the 4,771 MAGs using Prodigal v.2.6.3 (23) in single mode (-p single). The resulting protein sequences were functionally annotated by searching against the KEGG database using DIAMOND v.2.1.8.162 (24). The search parameters were set as follows: --min-score 60 --query-cover 70 --max-target-seqs 5 --masking 1. For each gene, the top-scoring match was retained as the final functional annotation.

### Identification of *de novo* vitamin-producing MAGs and phylogenetic profiling

To identify MAGs capable of *de novo* vitamin biosynthesis, the 4,771 MAGs were further screened for genome quality using CheckM2 v.1.0.1 (20). Only genomes with ≥90% completeness and <5% contamination were retained for in-depth analyses. Strain-level redundancy was minimized by dereplicating genomes with dRep v.3.4.5 (22) at ≥99% ANI, resulting in 2,682 representative genomes. Protein sequences were annotated using DIAMOND v.2.1.8.162 (24) via BLASTP searches against the KEGG database. Based on predefined sets of essential marker functions, 1,665 genomes were classified as putative vitamin producers capable of *de novo* synthesis of at least one vitamin. Specifically, the analysis covered eight B vitamins, thiamine, riboflavin, niacin, pantothenate, pyridoxine, biotin, folate, and cobalamin, as well as vitamin K_2_. For cross-host comparison, previously reported phylum-level distributions of vitamin-producing genomes in humans, chickens, cats, sheep, and Tibetan antelope were used as reference data (9, 11, 25). The mouse data were generated in the present study and were added to the previously established cross-host comparison.

### Infection impacts vitamins B and K_2_ biosynthesis in mice

Metagenomic data from PRJNA1419542 were reanalyzed, including samples from the AI, CI, and control groups. To ensure high-quality data, raw sequencing reads were processed with fastp v.0.23.0 (26) using the following parameters: -q 20 -u 30 -n 5 -y -Y 30 -l 80 --trim_poly_g. Quality-filtered reads were aligned to the mouse reference genome (NCBI RefSeq assembly GCF_000001635.27_GRCm39) using Bowtie2 v.2.5.0 (27) to remove host-derived sequences. The resulting clean reads were then mapped, again using Bowtie2 v.2.5.0, to the vitamin biosynthesis genome data set for downstream analyses. Mapped read counts were normalized to transcripts per million (TPM) to quantify the relative abundances of KOs and associated vitamin biosynthesis pathways. For taxonomic profiling, each vitamin biosynthesis-related gene inherited the taxonomy of its source MAG, which was classified using GTDB-Tk. TPM-normalized gene abundances were then summed by source-MAG taxonomy to estimate phylum- and genus-level contributions to vitamin biosynthesis.

To investigate the taxonomic sources and patterns of niacin (vitamin B_3_) biosynthesis, we examined the *de novo* niacin biosynthetic potential of the mouse gut microbiome through targeted analysis of niacin biosynthesis; core genes (*nadB*, *nadA*, *nadC*) were examined at the phylum level to resolve the taxonomic contributions of different microbial lineages to this pathway. TPM-normalized read counts were used both to compute the relative abundance of KOs and to summarize taxonomic contributions at the phylum and genus levels. For taxonomic profiling, the abundance of each taxon was calculated by summing the TPM values of all genes assigned to that taxon. KOs involved in vitamin biosynthesis pathways were considered functional markers, and their cumulative abundance was used to infer the relative abundance of the corresponding metabolic pathways.

### Statistical analyses and visualization

All statistical analyses were conducted in R (v.4.2.2). α-Diversity metrics, including the Simpson index and richness, were calculated from abundance matrices of both taxonomic features and functional gene sets to evaluate changes in the diversity and evenness of vitamin biosynthesis pathways between infected (AI and CI) and control mouse groups. Group-wise comparisons (infected vs control) of diversity indices, taxonomic profiles, and functional gene abundances were performed using the Wilcoxon rank-sum test. Between-sample variation was assessed using Bray-Curtis dissimilarities and visualized through principal coordinates analysis (PCoA). Overall group separation was evaluated with permutational multivariate analysis of variance. Other figures were generated using ggplot2 (v.4.0.2). Phylogenetic relationships of these vitamin-producing genomes were visualized using iTOL v.7.

## RESULTS

### Construction and taxonomic overview of the mouse gut genome catalog

**Fig 1:**
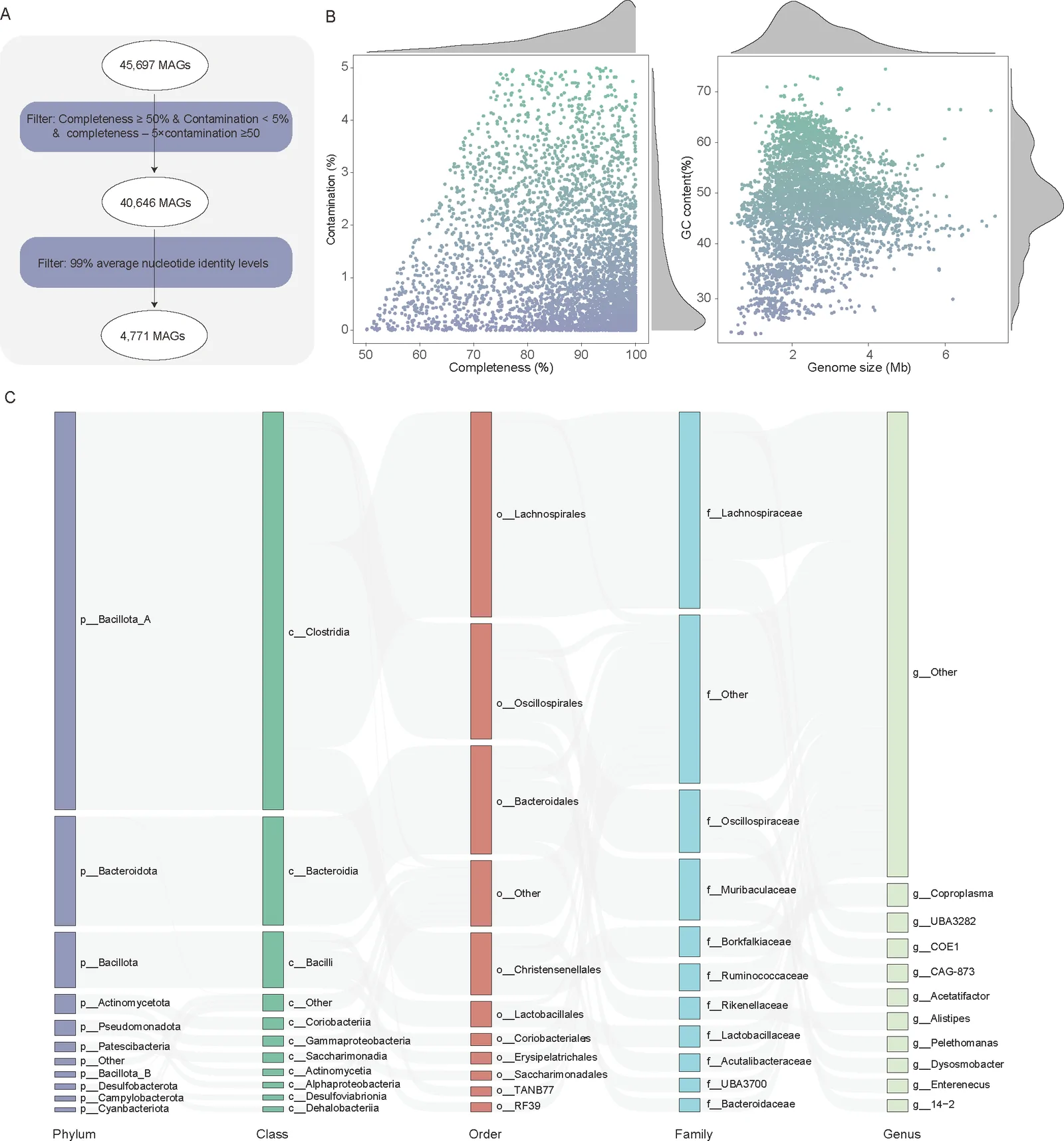
Construction and characterization of 4,771 non-redundant mouse gut MAGs. (**A**) Workflow for MAG quality filtering and dereplication. (**B**) Genome quality characteristics of the 4,771 representative MAGs, including completeness, contamination, N50, and N90 values. (**C**) GTDB-based taxonomic composition of the 4,771 representative MAGs. The panel summarizes the distribution of MAGs across major bacterial taxonomic groups. For each taxonomic rank, only the top 11 most abundant taxa are displayed to highlight the dominant microbial lineages within the mouse gut genome catalog.

To generate a comprehensive genome catalog of the mouse gut microbiome, we compiled 45,697 MAGs. These MAGs underwent stringent quality control, retaining only those with ≥50% completeness, <5% contamination, and a genome quality score (completeness − 5 × contamination) ≥50. Genomes were then dereplicated at a 99% ANI threshold, resulting in 4,771 non-redundant MAGs for downstream analyses (Fig. 1A). The retained MAGs ranged in size from 0.41 to 7.30 Mbp, with an average GC content of 48.42%. On average, genomes had 88.45% completeness and 1.14% contamination, with mean N50 and N90 values of 75.7 kbp and 35.9 kbp, respectively. Among these, 2,682 MAGs met a higher-quality standard, exhibiting >90% completeness and <5% contamination (Fig. 1B; Table S1).

GTDB-based taxonomic annotation revealed that the 4,771 MAGs spanned 19 phyla, 25 classes, 60 orders, 111 families, and 398 genera (Fig. 1C; Table S1). At the phylum level, the dominant lineages were Bacillota_A (62.8%, 2,997/4,771) and Bacteroidota (17.2%, 820/4,771), followed by Bacillota (8.8%, 420/4,771), Actinomycetota (3.0%, 144/4,771), and Pseudomonadota (2.4%, 116/4,771). At the family level, Lachnospiraceae (31.0%, 1,748/4,771), Oscillospiraceae (9.9%, 472/4,771), and Muribaculaceae (9.6%, 459/4,771) were the most prevalent. Additional families with notable abundance included Borkfalkiaceae (4.8%, 227/4,771), Ruminococcaceae (4.3%, 203/4,771), Rikenellaceae (4.3%, 203/4,771), Lactobacillaceae (3.5%, 167/4,771), and Acutalibacteraceae (3.3%, 158/4,771) (Table S1).

### Functional profiling of *de novo* vitamin B and K_2_ biosynthesis in the mouse gut

To investigate the contribution of the mouse gut microbiome to *de novo* vitamin biosynthesis, we identified 229,717 genes from the 4,771 MAGs and annotated them to 177 KOs. Functional annotation revealed that these genes are involved in the biosynthesis of eight B vitamins, thiamine, riboflavin, niacin, pantothenate, pyridoxine, biotin, folate, and cobalamin, as well as vitamin K_2_ (Table S2). A systematic survey of the alternative biosynthetic routes encoded in the mouse gut microbiome highlighted pathway-specific patterns. Thiamine biosynthesis can be supported by two distinct enzymatic pathways, whereas niacin biosynthesis occurs mainly via the *nadR*/*nadM* and NMNAT enzymatic branches.

Cobalamin, which is synthesized exclusively by prokaryotes, can be produced through either aerobic or anaerobic pathways (Fig. S1). Pantothenate biosynthesis relies on precursor branches that supply pantoate and β-alanine, which are subsequently condensed to form pantothenate and feed into the coenzyme A biosynthetic module. The pyridoxine pathway primarily utilizes intermediates derived from carbohydrate metabolism, while riboflavin biosynthesis shows a characteristic convergence of two precursor branches (Fig. S2). Biotin is synthesized via four independent biosynthetic branches, and menaquinone is produced through the futalosine pathway (Fig. S3). These results illustrate the diverse and modular enzymatic potential of the mouse gut microbiome to produce essential vitamins *de novo*, highlighting its critical role in maintaining host micronutrient availability.

### Genomic potential of vitamin biosynthesis in the mouse gut microbiome

**Fig 2:**
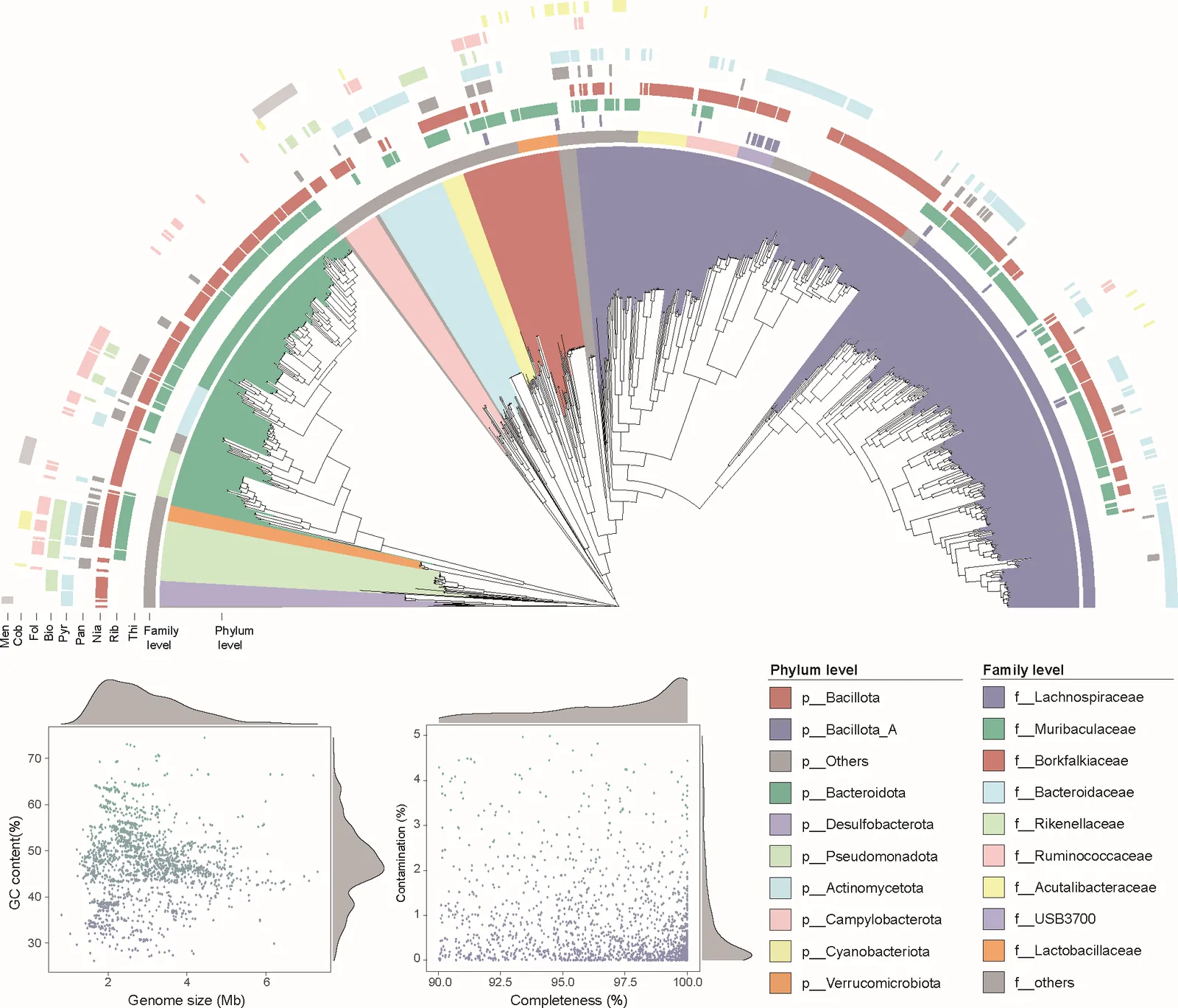
Phylogenetic and functional overview of 1,665 genomes capable of *de novo* synthesis of B vitamins and K_2_. A maximum-likelihood phylogenetic tree was constructed from genomes predicted to have vitamin biosynthetic capacity. The inner ring indicates phylum-level taxonomy, the first outer ring shows family-level classification, and outer rings 3–11 depict the presence (colored) or absence (blank) of biosynthetic capacity for each vitamin: thiamine (Thi), riboflavin (Rib), niacin (Nia), pantothenate (Pan), pyridoxine (Pyr), biotin (Bio), folate (Fol), cobalamin (Cob), and menaquinone (Men). The genome size–GC content scatter plot shows the distribution of genome size and GC content among the 1,665 genomes. The completeness–contamination scatter plot shows the genome quality distribution of these genomes.

To further assess the genomic potential for *de novo* vitamin B and vitamin K_2_ biosynthesis, we selected a high-quality subset from the 4,771 non-redundant representative MAGs using stricter criteria of completeness ≥90% and contamination <5%, followed by dereplication at a 99% ANI threshold. This filtering resulted in 2,682 high-quality genomes. Functional annotation showed that 1,665 of these genomes encoded complete *de novo* biosynthetic pathways for at least one B vitamin or vitamin K_2_ and were therefore considered putative vitamin-producing genomes (Table S3). The high-quality genomes had an average size of 2.90 Mbp and a mean GC content of 47.12% (Fig. 2). Among the genomes with predicted vitamin biosynthetic capacity, 726 could produce only a single vitamin, whereas 870 could synthesize between two and four vitamins. No genome was able to produce all eight B vitamins, and only eight genomes exhibited the capacity to synthesize seven vitamins, including three assigned to *Klebsiella* and two to *Bacillus*. These results suggest pronounced functional specialization and metabolic complementarity in *de novo* vitamin biosynthesis within the mouse gut microbiome (Table S4).

When examining individual vitamins, the number of genomes predicted to contribute to their biosynthesis varied widely: thiamine (47 genomes), riboflavin (910), niacin (1,150), pantothenate (264), pyridoxine (529), biotin (140), folate (186), cobalamin (80), and vitamin K2 (61). The uneven distribution of vitamin biosynthetic pathways across genomes underscores a community-level mechanism in which cooperative biosynthesis among multiple taxa collectively fulfills the host’s vitamin requirements (Table S4). Bacillota_A emerged as the primary driver of vitamin biosynthesis in the mouse gut microbiome, contributing 52.3% of the predicted capacity. When the mouse data generated in the present study were compared with previously reported host-specific data, marked differences in the dominant phyla contributing to vitamin biosynthesis were observed across hosts. Bacillota_A contributed 50.4% in humans, Bacteroidota (28.2%) and Bacillota_A (25.7%) jointly dominated in chickens, Bacillota_A accounted for 40.5% in cats and sheep, and 45.1% in Tibetan antelope. These patterns indicate that the vitamin biosynthetic potential of gut microbiomes is strongly host-specific, reflecting ecological adaptation (Fig. S4).

### Stage-dependent disruption of microbial vitamin biosynthesis during *T. gondii* infection

To evaluate infection-associated changes in microbial vitamin biosynthesis, we analyzed 30 mouse gut shotgun metagenomic samples from NCBI BioProject PRJNA1419542. Quality-filtered reads were mapped to the curated mouse gut vitamin biosynthesis genome data set, and TPM-normalized abundances of vitamin biosynthesis-related KOs were used to calculate pathway-level abundance profiles and diversity metrics.

**Fig 3:**
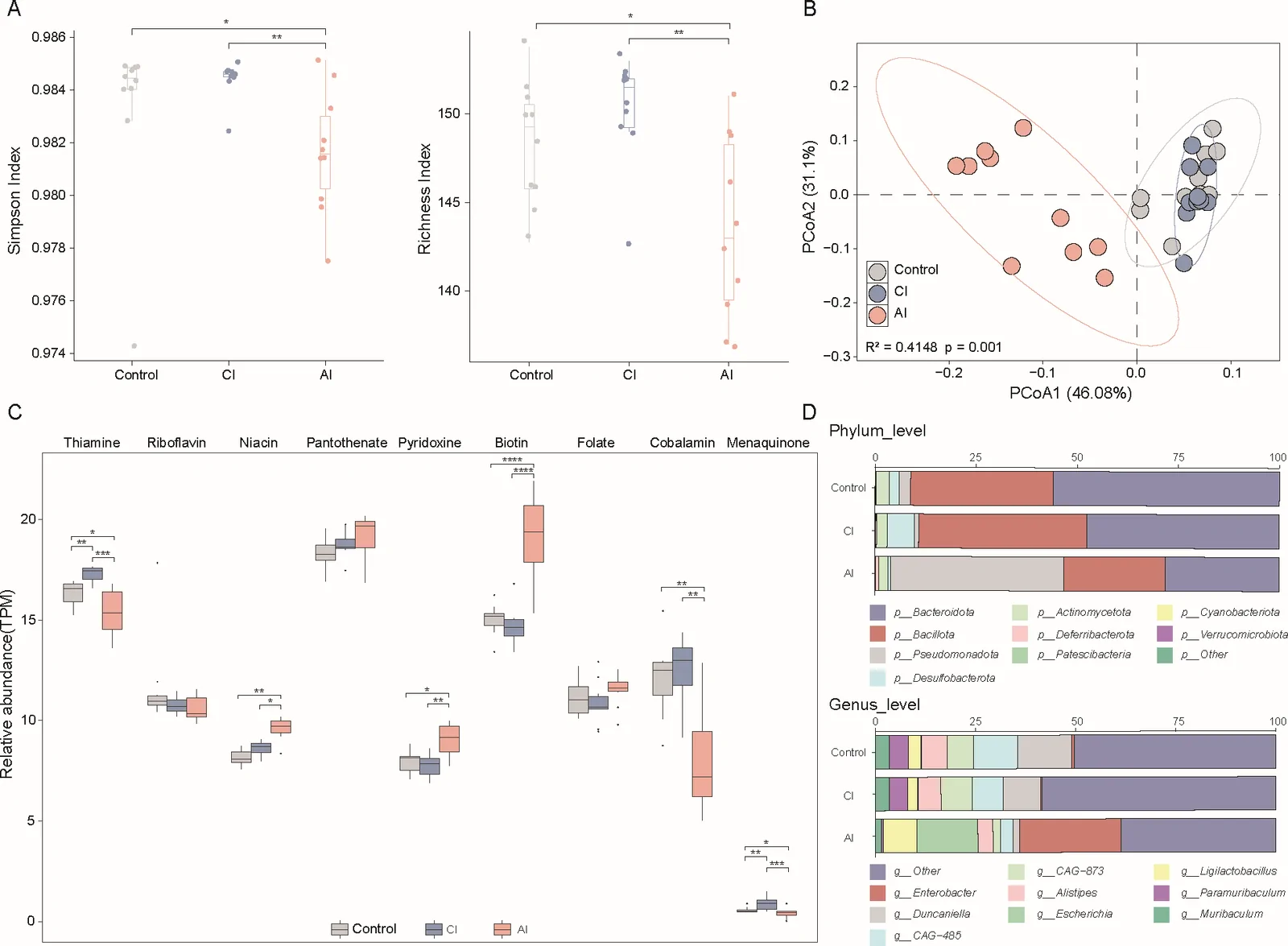
Diversity, abundance, and taxonomic distribution of vitamin B and K_2_ biosynthesis-related functions across different stages of *T. gondii* infection. (**A**) α-Diversity of vitamin biosynthesis-related KOs, calculated from TPM-normalized KO abundance profiles using the Simpson index (left) and richness (right). Control, CI, and AI denote control, chronic, and acute infection groups, respectively. Statistical significance was evaluated using the Wilcoxon rank-sum test (*P < 0.05; **P < 0.01). (**B**) PCoA based on Bray-Curtis dissimilarities calculated from TPM-normalized abundance profiles of vitamin biosynthesis-related KOs. Points represent individual samples, and ellipses indicate 95% confidence intervals for each group. (**C**) TPM-normalized relative abundances of vitamin biosynthesis-related KOs grouped according to the nine vitamin biosynthetic pathways. Statistical differences among groups were evaluated using the Wilcoxon rank-sum test (*P < 0.05; **P < 0.01; ***P < 0.001). (**D**) Phylum- and genus-level taxonomic distributions of vitamin biosynthesis-related genes, determined according to the taxonomy of their source MAGs and weighted by TPM-normalized gene abundances.

Analysis of gut metagenomic data revealed that acute *T. gondii* infection significantly reduced α-diversity (i.e., overall vitamin biosynthetic capacity) of vitamin B and K_2_ biosynthetic pathways compared with CI and control groups (Simpson index, *P* < 0.01; Fig. 3A). β-Diversity analysis further demonstrated clear separation of acute infection from other groups in the overall structure of vitamin biosynthesis pathways (PCoA, Fig. 3B). Focusing on individual vitamins, six of nine pathways exhibited significant differences in the relative abundances of KOs (Fig. 3C). Taxonomic profiling of vitamin biosynthesis genes revealed that four phyla and five genera exhibited significant differences between the infected (AI/CI) and control groups (Fig. 3D).

**Fig 4:**
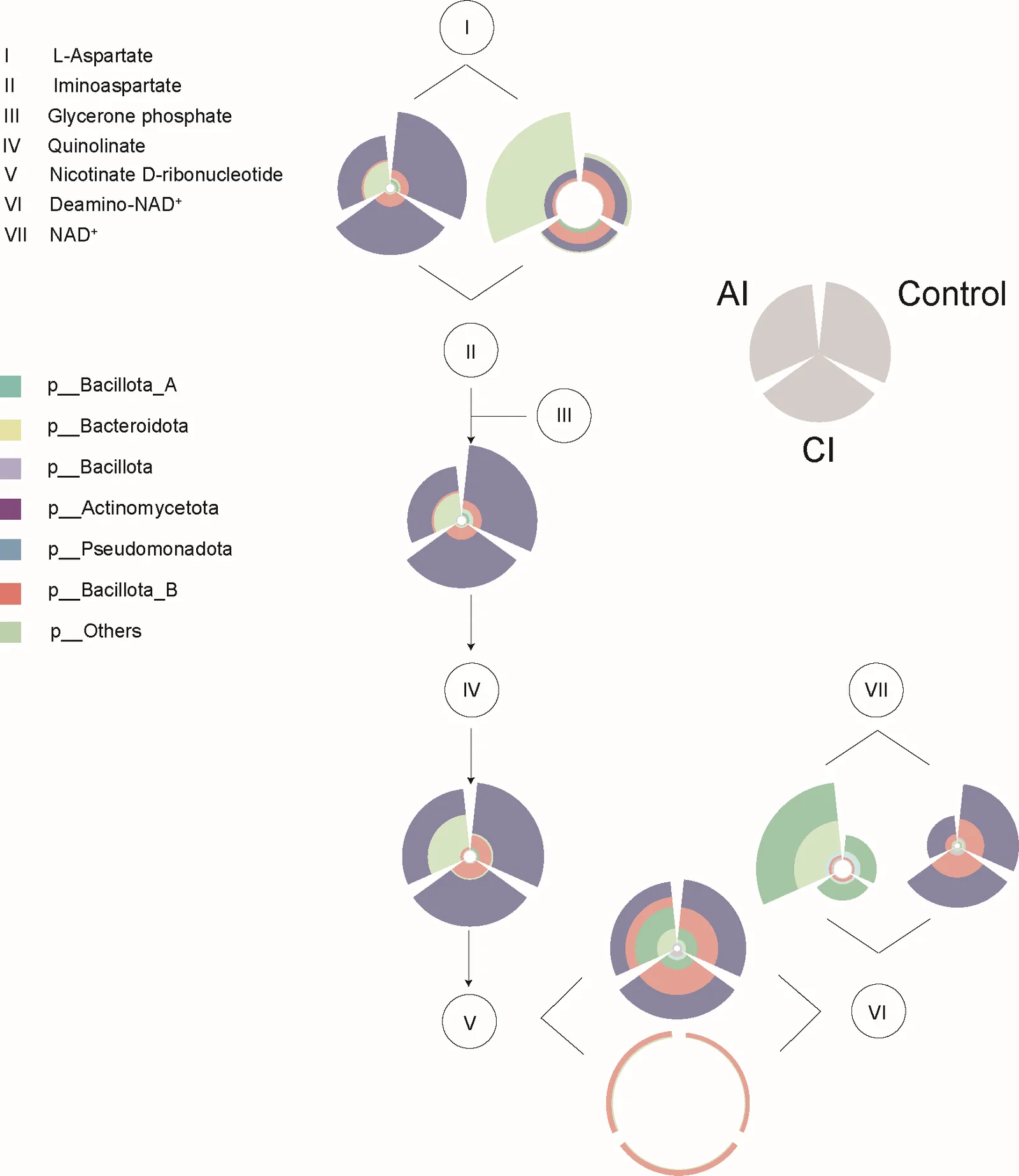
Niacin biosynthesis pathway and its dynamics across infection stages. The pathway comprises eight enzymatic functions and six intermediate metabolites, represented as rectangles. The circular heatmap shows phylum-level distributions across control, CI, and AI groups.

Among the vitamin biosynthesis pathways showing infection-associated changes, niacin (vitamin B_3_) was further examined because of its direct connection to NAD^+^ and NADP^+^ metabolism, which is closely related to redox homeostasis, energy metabolism, and immune responses during infection. The abundances of niacin-related KOs were significantly higher in the AI group compared with the CI and control groups. Taxonomic profiling revealed that phylum-level and genus-level distributions of niacin-related genes were significantly altered in acute infection, highlighting the ecological restructuring of vitamin-producing taxa under host immune and metabolic pressures (Fig. 3D; Fig. S5). While overall vitamin pathway diversity declined during acute infection, the relative abundance of core niacin biosynthesis genes (*nadB*, *nadA*, *nadC*) increased and displayed a marked phylum-level redistribution (Fig. 4). These genes encode key enzymatic steps in the *de novo* NAD^+^ biosynthetic pathway, suggesting selective remodeling of microbial NAD^+^ precursor production capacity during acute infection.

Other redox-relevant vitamins, including riboflavin and pantothenate, showed less pronounced shifts, indicating a preferential modulation of the niacin/NAD^+^ axis. This selective remodeling suggests a targeted microbial response to infection-induced metabolic stress, potentially linked to host NAD^+^ demand. These results highlight infection stage-dependent shifts in both the functional potential and taxonomic contributors of niacin biosynthesis, providing new insights into the microbial contributions to host vitamin metabolism during *T. gondii* infection.

## DISCUSSION

The gut microbiome plays a central role in host nutrition and metabolic homeostasis, particularly through the *de novo* biosynthesis of essential vitamins such as B vitamins and vitamin K_2_ (11, 28, 29). How *T. gondii* infection influences the vitamin biosynthetic potential of the microbiome remains unclear. In this study, we provide a genome-resolved perspective on microbial vitamin biosynthesis in the mouse gut and its modulation by *T. gondii* infection, with a particular emphasis on the niacin/NAD^+^ axis. Using 4,771 representative MAGs and high-quality subsets, we mapped *de novo* biosynthetic potential for eight B vitamins and vitamin K_2_, identifying 1,665 genomes capable of synthesizing at least one vitamin independently. Our analysis confirms the functional specialization and community-level complementarity of microbial vitamin networks, which likely buffer host vitamin availability under normal conditions. Similar genome-resolved approaches have also been used to reveal successional dynamics and functional transitions of gut microbial communities across host developmental stages, further supporting the value of genome-resolved analyses for linking microbial lineages with functional potential (30).

The mouse gut genome catalog revealed an extensive repertoire of genes involved in vitamin biosynthesis, highlighting the substantial metabolic capacity of gut microbial communities to contribute to host micronutrient homeostasis. Functional profiling demonstrated that the murine gut microbiome harbors diverse genes supporting the *de novo* biosynthesis of eight B vitamins and vitamin K_2_, indicating that microbial communities constitute an important reservoir of biosynthetic potential complementing host nutritional requirements (31). This observation aligns with the concept that gut microbes function as a metabolically integrated extension of the host, supplying small bioactive molecules and essential cofactors, including vitamin precursors, that support both host metabolic processes and microbial communities (8, 32). In addition to their abundance, vitamin biosynthetic pathways displayed considerable biochemical diversity. The coexistence of alternative enzymatic routes for thiamine and niacin biosynthesis suggests that different microbial taxa employ distinct biochemical strategies to generate the same end products (33).

Consistent with this metabolic flexibility, several vitamin biosynthesis pathways exhibited modular organization linking them to central metabolic processes (10). For example, pyridoxine biosynthesis draws intermediates from central carbohydrate metabolism, while riboflavin and biotin pathways incorporate precursor branches derived from diverse metabolic inputs. These architectures highlight how vitamin biosynthesis is embedded within broader metabolic networks that connect energy metabolism, nutrient availability, and microbial growth. The presence of both aerobic and anaerobic cobalamin biosynthesis pathways further underscores the ecological diversity of microbial contributors to vitamin production. Because cobalamin synthesis is restricted to prokaryotes and functions as a cofactor in numerous reactions related to carbon metabolism and fermentation, its production within the gut microbiome may influence metabolic interactions among microbial taxa (34). The observed reduction in microbial cobalamin biosynthetic potential during acute infection may, therefore, reflect disruption of bacterial taxa involved in carbon metabolism, fermentation, and microbial cross-feeding (35, 36). From the host perspective, this change could affect microbiome-derived cofactor availability related to energy metabolism and mucosal immune homeostasis (37, 38), whereas from the parasite perspective, it may alter the local nutritional environment during infection. However, because vitamin concentrations, host physiological responses, and parasite burden were not directly measured, this interpretation remains hypothesis-generating.

Beyond cobalamin, other cofactor biosynthetic pathways further illustrate the functional specialization of gut microbial metabolism. The detection of menaquinone biosynthesis through the futalosine pathway highlights the role of gut microbes in generating cofactors associated with anaerobic respiration and electron transport. Menaquinone derivatives are central to microbial energy metabolism (39) and have also been implicated in key physiological processes, including bone metabolism and cardiovascular health (40), suggesting additional routes through which microbial metabolism may influence host physiology.

Vitamin biosynthetic potential was unevenly distributed across microbial lineages within the mouse gut. Although genes for the biosynthesis of eight B vitamins and vitamin K_2_ were detected across the catalog, most genomes encoded capacity for only a subset of these pathways. This pattern reflects strong functional specialization and metabolic complementarity among community members, suggesting that vitamin availability in the gut environment is sustained through cooperative interactions among multiple taxa (16, 41). In complex microbial ecosystems, many organisms lack complete biosynthetic pathways and instead rely on metabolites released by neighboring species, forming trophic interaction networks that structure microbial community assembly and function (10, 42). Such distributed metabolic architectures may enhance gut microbial resilience by allowing essential biosynthetic processes to persist even when individual taxa decline (43).

Against this ecological backdrop, infection-driven perturbations provide an opportunity to examine how cooperative microbial biosynthetic networks respond to host physiological stress. Acute *T. gondii* infection was associated with reduced diversity of vitamin biosynthesis-related pathways and altered niacin biosynthesis profiles. Core niacin biosynthesis genes (*nadB*, *nadA*, and *nadC*) exhibited phylum-specific redistribution, with increased relative abundance in specific Bacillota_A lineages. Shifts in the taxonomic contributors to niacin biosynthesis suggest that certain microbial taxa may possess adaptive advantages under inflammatory conditions. Consistent with previous studies (9), our results indicate that host infection reshapes not only the taxonomic structure of the gut microbiome but also the distribution of metabolic functions involved in nutrient and cofactor availability.

Niacin biosynthesis represents a key metabolic node because it supplies precursors for NAD^+^ and NADP^+^, essential cofactors involved in redox reactions, energy metabolism, and immune activation (13, 14). The widespread occurrence of niacin biosynthesis genes likely reflects the importance of these cofactors in microbial and host metabolism. Rather than causing a uniform loss of biosynthetic potential, infection appears to reshape the distribution of pathways linked to redox homeostasis (15). Infection-associated dysbiosis has previously been reported to persist throughout chronic *T. gondii* infection, with expansion of several inflammatory-associated microbial families and reductions in beneficial commensals (4), which may contribute to the functional redistribution of vitamin biosynthesis pathways observed in our analysis.

Acute *T. gondii* infection is characterized by a strong IL-12–driven Th1 inflammatory response with high levels of IFN-γ and systemic metabolic stress, including hypermetabolism and weight loss, reflecting increased metabolic demand in the host (44–46). These conditions accelerate NAD^+^ turnover in host tissues through increased energy demand and activation of NAD-consuming enzymes (14). Previous organ-resolved metabolomic analyses of *T. gondii* infection demonstrated widespread metabolic perturbations across multiple organs during the acute stage, including the liver, heart, small intestine, muscle, and cecum, with changes most strongly associated with host immune responses and shifts in commensal microbial communities rather than local parasite burden (4). Within this context, the restructuring of microbial niacin biosynthesis observed in our study may reflect ecological selection for taxa capable of sustaining NAD^+^ precursor production under inflammatory conditions, thereby contributing to host–microbiome metabolic crosstalk during infection.

Although host NAD^+^ levels were not directly measured in the present study, the redistribution of microbial NAD^+^ precursor pathways links infection-driven microbiome remodeling with host redox metabolism. Other NAD^+^-linked vitamins, including riboflavin and pantothenate, exhibited less pronounced stage-specific changes, suggesting that the NAD^+^ axis may be particularly responsive to infection-associated metabolic and immune stress.

Functional redundancy within microbial communities likely buffers NAD^+^ precursor availability, suggesting that community-level complementarity mitigates acute disruptions in vitamin biosynthetic capacity (43, 47). Consistent with this buffering capacity, chronic infection was associated with partial recovery of vitamin pathway diversity, indicating adaptive stabilization of microbial functional potential as host–microbiome interactions re-equilibrate over time. Supporting this observation, organ-resolved metabolomics has shown that metabolic perturbations are most widespread during acute infection but become more localized to the gastrointestinal tract during chronic infection, paralleling the recovery of microbial vitamin biosynthetic diversity observed in our data set (4). More broadly, the uneven representation and differential infection responses of vitamin biosynthetic pathways suggest that individual vitamins occupy distinct ecological roles within the microbial community. Because overall taxonomic richness and functional richness were not systematically compared in this study, the observed changes in vitamin biosynthesis, including vitamin K_2_-related pathways, may partly reflect broader microbiome perturbation during acute infection.

Other nutrient-related pathways may also contribute to host–parasite interactions during *T. gondii* infection. For example, purines (48), sterol/cholesterol-related metabolites (49), tryptophan (50), and arginine (51) are important for parasite growth, survival, and host immune responses. Although these pathways are highly relevant, the present study was specifically designed to characterize genome-resolved microbial vitamin B and vitamin K_2_ biosynthetic potential, with particular emphasis on niacin/NAD^+^-related functions. Therefore, broader nutrient metabolism pathways relevant to *T. gondii* growth were not systematically analyzed here. Future studies integrating metagenomics, metatranscriptomics, and metabolomics will be needed to determine whether microbiome-derived purine, sterol/cholesterol-related, tryptophan, and arginine metabolism contributes to parasite growth restriction or host resistance during infection.

### Conclusion

We constructed a comprehensive genome-resolved catalog of the mouse gut microbiome and characterized its potential for *de novo* biosynthesis of eight B vitamins and vitamin K_2_ Analysis of 4,771 representative MAGs revealed extensive microbial capacity for vitamin production and highlighted functional specialization and metabolic complementarity among microbial taxa. Examination of metagenomic data from *T. gondii*–infected mice demonstrated stage-dependent disruptions in microbial vitamin biosynthesis, with acute infection associated with reduced diversity of vitamin pathways and phylum-specific shifts in key niacin biosynthesis genes (*nadB*, *nadA*, *nadC*). These findings show that *T. gondii* infection reshapes not only microbiome composition but also microbial functions involved in host nutrient metabolism. Overall, our results provide a genome-resolved framework linking microbial taxa to vitamin biosynthetic potential in the mouse gut and identify niacin biosynthesis as a responsive pathway during infection. This work lays a foundation for future studies exploring microbiome-mediated strategies to support host vitamin homeostasis during infection.

## Supplementary Material

Reviewer comments

## Data Availability

The CMMG data set is accessible from https://ezmeta.unige.ch/CMMG. The 3,899 MAGs and the shotgun metagenomic samples analyzed in this study are available in the NCBI database under BioProject accession number PRJNA1419542.

## SUPPLEMENTAL MATERIAL

Supplemental figures spectrum.01564-26-s0001.docx Fig. S1 to S5.

Tables S1 to S4 spectrum.01564-26-s0002.xlsx Table S1: Overview of 4,771 nonredundant representative metagenome-assembled genomes (MAGs) from the mice gut microbiome. Table S2: KEGG-based functional annotation of microbial genes associated with B-vitamin and vitamin K_2_ biosynthesis pathways. Table S3: Overview of vitamin biosynthetic potential and taxonomic classification across 2,682 high-quality genomes. Table S4: Overview of 1,665 high-quality genomes confirmed to independently complete *de novo* biosynthesis of at least one vitamin.

OPEN PEER REVIEW reviewer-comments.pdf An accounting of the reviewer comments and feedback.

ASM does not own the copyrights to Supplemental Material that may be linked to, or accessed through, an article. The authors have granted ASM a non-exclusive, world-wide license to publish the Supplemental Material files. Please contact the corresponding author directly for reuse.
