# Supplementary material for: Genome-resolved analysis reveals disruption of gut microbial vitamin B and K2 biosynthesis during Toxoplasma gondii infection in mice
Source: Microbiol Spectr. 2026 Sep 8;14(10):e01564-26. doi: 10.1128/spectrum.01564-26 (PMC13688803; doi:10.1128/spectrum.01564-26)
Supplement: Supplementary file 1 [file reviewer-comments.pdf]

ASM | Microbiology Spectrum

Genome-resolved analysis reveals disruption of gut microbial vitamin B and K_2 biosynthesis during *Toxoplasma gondii* infection in mice

Hai-Long Yu, Hany M. Elsheikha, Hai-Ping Wang, Ying-Qian Gao, Rui Liu, He Ma, Jing Jiang, Ying Li, and Xiao-Xuan Zhang

Corresponding Author(s): Xiao-Xuan Zhang, College of Veterinary Medicine, Qingdao Agricultural University, Qingdao, Shandong Province, PR China.

Review Timeline:

Submission Date:	May 7, 2026
Editorial Decision:	June 19, 2026
Revision Received:	July 13, 2026
Editorial Decision:	August 7, 2026
Revision Received:	August 12, 2026
Accepted:	August 17, 2026

Editor: Zhicheng Dou

Reviewer(s): The reviewers have opted to remain anonymous.

Transaction Report:

(Note: With the exception of the correction of typographical or spelling errors that could be a source of ambiguity, letters and reports are not edited. The original formatting of letters and referee reports may not be reflected in this compilation.)

DOI: https://doi.org/10.1128/spectrum.01564-26

Re: Spectrum01564-26 (**Genome-resolved analysis reveals disruption of gut microbial vitamin B and K_2 biosynthesis during *Toxoplasma gondii* infection in mice**)

Dear Prof. Xiao-Xuan Zhang:

Thank you for the privilege of reviewing your work. Below you will find my comments, instructions from the Spectrum editorial office, and the reviewer comments.

Please return the manuscript within 60 days; if you cannot complete the modification within this time period, please contact me. If you do not wish to modify the manuscript and prefer to submit it to another journal, notify me immediately so that the manuscript may be formally withdrawn from consideration by Spectrum.

Revision Guidelines
To submit your modified manuscript, log into the submission site at https://spectrum.msubmit.net/cgi-bin/main.plex. Go to Author Tasks and click the appropriate manuscript title to begin. The information you entered when you first submitted the paper will be displayed; update this as necessary. Note the following requirements:

• Upload point-by-point responses to the issues raised by the reviewers in a file named "Response to Reviewers," NOT in your cover letter.
• Upload a compare copy of the manuscript (without figures) as a "Marked-Up Manuscript" file.
• Upload a clean .DOC/.DOCX version of the revised manuscript and remove the previous version.
• Each figure must be uploaded as a separate, editable, high-resolution file (TIFF or EPS preferred), and any multipanel figures must be assembled into one file.
• Any supplemental material intended for posting by ASM should be uploaded with their legends separate from the main manuscript. You can combine all supplemental material into one file (preferred) or split it into a maximum of 10 files with all associated legends included.

For complete guidelines on revision requirements, see our Submission and Review Process webpage. Submission of a paper that does not conform to guidelines may delay acceptance of your manuscript.

Data availability: ASM policy requires that data be available to the public upon online posting of the article, so please verify all links to sequence records, if present, and make sure that each number retrieves the full record of the data. If a new accession number is not linked or a link is broken, provide Spectrum production staff with the correct URL for the record. If the accession numbers for new data are not publicly accessible before the expected online posting of the article, publication may be delayed; please contact production staff (Spectrum@asmusa.org) immediately with the expected release date.

Publication Fees: For information on publication fees and which article types are subject to charges, visit our website. If your manuscript is accepted for publication and any fees apply, you will be contacted separately about payment during the production process; please follow the instructions in that e-mail. Arrangements for payment must be made before your article is published.

ASM Membership: Corresponding authors may join or renew ASM membership to obtain discounts on publication fees. Need to upgrade your membership level? Please contact Customer Service at Service@asmusa.org.

The ASM Journals program strives for constant improvement in our submission and publication process. Please tell us how we can improve your experience by taking this quick Author Survey.

Thank you for submitting your paper to Spectrum.

Sincerely,
Zhicheng Dou
Editor
Microbiology Spectrum

Reviewer #1 (Public repository details (Required)):

Maybe? Unclear if all data was from previously generated studies; there is only 1 BioProject listed, although possibly data from 2 studies were used.

Reviewer #1 (Comments for the Author):

Yu et al present data comparing microbial vitamin K pathways during Toxoplasma gondii infection. T. gondii-microbiota interactions are likely understudied; given infection and potential host effects of any infection, understanding how the gut microbiota might modulate infection is of interest. Overall, focus on a particular functional process, such as vitamin K metabolism, could also be of interest to the field. Building a mouse MAG database, then mapping metagenomic data from an actual study is a creative comparison and could also be a useful platform for other MAG-based studies. However, it is difficult to assess how well the presented data supports the study conclusions (i.e., vit K pathway diversity decreases during acute T. gondii infection), how this type of bioinformatic method could be replicated, or how important this might be from the manuscript in its current form. The relevance of vitamin K metabolism and T. gondii is never rationalized. The manuscript seemingly uses metagenomic data from a previously conducted study; while this is fine, study design, results concerning general microbiota differences, or vitamin K importance are never described to give context to the actual infection group. Methods (both in materials/methods as well as in legends) are inadequately described, which makes it difficult to assess study rigor. Which MAG groups and data are actually used in each of the analyses is also difficult to understand. Finally, the results and presented analyses do not always seem to match each other, which further decreases from being able to assess rigor or importance.
Specific comments below:
1) Throughout the manuscript, additional context about the study and what data was used in specific comparisons is lacking. This makes it difficult to assess whether the analyses support author conclusions.
- The introduction is not very clear in the study design, rationale, or aims. For instance, why focus specifically on vitamin K biosynthesis in the first place? What concrete microbiota changes have been previously established during this infection? Section is relatively vague.
- Overall, figure legends do not comprehensively explain figures. For instance, what is Figure 1C trying to display? This does not seem to demonstrate percentages across all MAGs, as described in the results section. Figure 2 has panels that are not described. Figure 3 (nor the associated results) does not specify what data specifically was used to calculate different diversity metrics.
- Similarly, the described results do not match up to what is visualized in the Figures. Overall, the figures themselves are not very rigorous in supporting the results.
- Throughout the manuscript, it is unclear what data is being used. Dereplication, QC filtering, and functional profiling seems to be done multiple times for vitamin K. For example, first for 4771 MAGs (line 226+), then again for 2682 MAGs (line 248+). Where are these MAGs coming from? Is random mouse metagenomic data / MAGs used, or are these analyses from a (potentially previous) mouse toxoplasma study?
- (Figure 3) Which MAG sets, or what metagenomic data, were actually used to describe the mouse toxoplasma study results? How was this model set up? Context is needed.
- There is no description of infection stages, so it is difficult to assess importance of microbiota comparisons between these 'groups' (lines 271+). Importantly, what kinds of samples this metagenomic sequencing was extracted from is never described.
- What were overall microbiota differences across the infection groups? Does vit K differences alone account for this? If general microbiota richness is also decreased during acute infection, the decrease in vit K might just be a reflection of this. Would suggest showing taxonomic / overall functional microbiota richness across the different groups, then comparing whether vit K pathways specifically differ from these trends.
- How exactly was taxonomic profiling of vit K synthesis genes conducted compared in Figure 3D?
- (lines 283 - 289) Why was niacin singled out here, as other vit K pathways also appear to display significantly higher abundances? Relatedly, Figure 3D does not appear to support the associated statement in the results.

2) Many of the methods are inadequately described.
- MAG generation from samples collected in current study are insufficiently described. What programs were used to do this?
- Were toxoplasma-associated metagenomic data also from previously deposited info, or generated in this study? There is 1 reference (#18) from which most MAGs were built from, but this appears to be just to set up a database. There is also another study (possibly?) from which toxoplasma-specific datasets were derived from, but no reference is given. However, public repository information is given here. If from a previous study, some additional description of the study design would also be helpful for context.
- A supplemental table specifying the MAGs generated as part of this study, as well as which specific previously deposited MAGs were actually used in the study and their associated metadata (i.e., taxonomic classification, specific accession number, MAG QC info, etc), would be helpful.
- Figures seems to contain more analyses (phylogenetic trees, etc) that are never described in Methods.

Reviewer #2 (Comments for the Author):

This paper aims to detect changes in the mouse microbiome following acute and chronic infections by Toxoplasma parasites. By analyzing microbiome data from these conditions, they found that Toxoplasma infections disrupt microbial vitamin biosynthesis in mice; specifically, acute infection reduces the diversity of vitamin pathways. A few comments are listed below.

1. How does the microbiome change upon Toxoplasma infections alter the mouse physiology and nutrient perspective? Or can the changes help the host resist Toxoplasma infection? Although the authors stated a few scenarios in the discussion, why are they important for the host to adapt to this change? For example, how does the reduction of cobalamin production during acute infection affect the infection from the parasite and/or host sides?

2. Besides nutrient genes, it would be more interesting to see if other essential nutrient production for Toxoplasma gets changed upon interaction, for example, purines, cholesterol, tryptophan, and arginine, which are essential nutrients for Toxoplasma growth. Reducing the production of these nutrients in the mouse microbiome could limit parasite growth. Therefore, the broad view of these nutrient metabolism genes from the comparison would strengthen the manuscript.

3. Line 87 has a typo. It should be "linked."

Re: Revised Manuscript ID Spectrum01564-26

Dear Editors and Reviewers

On behalf of all authors, I would like to thank the Editor and two reviewers for their comments and constructive suggestions on our **Manuscript ID Spectrum01564-26**. We made major changes to the manuscript based on the reviewers' comments, corrected all errors, added experimental details, supplemented data, and optimized the presentation. In accordance with the reviewers' suggestions, we have thoroughly revised the manuscript by addressing all identified errors, incorporating additional details on data collection and analysis, and refining the overall presentation for improved clarity. In the following section, we detail our point-by-point responses to the specific comments and suggestions.

Authors' replies to reviewers' comments

Responses to the comments and suggestions of reviewer #1:

Comment 1:

Maybe? Unclear if all data was from previously generated studies; there is only 1 BioProject listed, although possibly data from 2 studies were used.

Response: We thank the reviewer for pointing out this issue. To address this concern, we have revised the **Data availability section** to explicitly clarify the origins of all datasets analyzed in this study **(Line 483~485)**.

Comment 2:

Throughout the manuscript, additional context about the study and what data was used in specific comparisons is lacking. This makes it difficult to assess whether the analyses support author conclusions.

The introduction is not very clear in the study design, rationale, or aims. For instance, why focus specifically on vitamin K biosynthesis in the first place? What concrete microbiota changes have been previously established during this infection? Section is relatively vague.

Response: We thank the reviewer for this helpful comment. To address this

concern, we revised the Introduction to more clearly explain the rationale for focusing on microbial B-vitamin and vitamin K_2 biosynthetic pathways, emphasizing their roles as microbiome-derived metabolic functions relevant to host metabolism, microbial physiology, redox balance, and microbial electron transport. We also clarified that the study was not focused exclusively on vitamin K biosynthesis, but systematically examined eight B vitamins and vitamin K_2, with particular attention to niacin/NAD^+ metabolism because of its infection-stage-associated remodeling .

In addition, we added clearer descriptions of the study design and datasets in the Materials and Methods section. Specifically, we clarified that the study consisted of two analytical components: construction of a non-redundant mouse gut MAG catalog and reanalysis of shotgun metagenomic data from control, acute infection, and chronic infection groups **(Line135~144)**. These revisions provide clearer context for the analyses and improve the connection between the data, results, and conclusions.

Comment 3:

Overall, figure legends do not comprehensively explain figures. For instance, what is Figure 1C trying to display? This does not seem to demonstrate percentages across all MAGs, as described in the results section. Figure 2 has panels that are not described. Figure 3 (nor the associated results) does not specify what data specifically was used to calculate different diversity metrics.

Response: We thank the reviewer for this helpful comment. We agree that the previous figure legends were not sufficiently detailed. We have revised the legends for Figs. 1–3 and the associated Results section to improve clarity.

Specifically, we rewrote the legend for Fig. 1C to clarify that it shows the GTDB-based taxonomic composition of the 4,771 representative MAGs, and we added **Supplementary Table S1** (**Line 240)** to provide detailed taxonomic information supporting the percentages reported in the Results. We also expanded the Fig. 2 legend to describe the phylogenetic tree, taxonomic rings, vitamin biosynthetic capacity rings, and lower scatter plots showing genome size, GC content, completeness, and contamination (**Line 509~512)**. For Fig. 3, we revised the Results

section to specify that the diversity analyses were based on 30 mouse gut shotgun metagenomic samples from PRJNA1419542, including AI, CI, and control groups with 10 samples per group, and that TPM-normalized abundances of vitamin biosynthesis-related KOs were used for the calculations (**Line 291~295)**.

Comment 4:

Similarly, the described results do not match up to what is visualized in the Figures. Overall, the figures themselves are not very rigorous in supporting the results.

Response: We thank the reviewer for this important comment. We have carefully rechecked the consistency between the Results text and the information presented in the figures. We revised the relevant Results descriptions, figure legends, and supplementary information to ensure that the figures accurately support the reported findings.

Comment 5:

Throughout the manuscript, it is unclear what data is being used. Dereplication, QC filtering, and functional profiling seems to be done multiple times for vitamin K. For example, first for 4771 MAGs (line 226+), then again for 2682 MAGs (line 248+). Where are these MAGs coming from? Is random mouse metagenomic data / MAGs used, or are these analyses from a (potentially previous) mouse toxoplasma study?

Response: We thank the reviewer for pointing out this important issue. We agree that the previous description did not sufficiently clarify the relationship among the different MAG sets and the datasets used for each analysis.To address this concern, we have revised the Materials and Methods and Results sections to clarify the study design and data sources **(Line 135~145).** Specifically, the 4,771 MAGs represent the non-redundant mouse gut genome catalog constructed from 45,697 MAGs after quality filtering based on contamination <5%, completeness ≥50%, and a genome quality score (completeness − 5 × contamination) ≥50, followed by dereplication at a

99% ANI threshold **(Line 227~236)**.

The 2,682 MAGs represent a high-quality subset of this 4,771-MAG catalog, selected using more stringent criteria of completeness ≥90% and contamination <5%. This subset was used for in-depth identification of genomes with complete de novo biosynthetic pathways for eight B vitamins and vitamin K_2. Thus, the dereplication, quality filtering, and functional profiling were not separate or duplicated analyses for vitamin K only, but sequential steps applied at different stringency levels for different analytical purposes **(Line 270~276)**.

We also clarified that infection-associated comparisons were performed using 30 mouse gut shotgun metagenomic samples from PRJNA1419542, including acute T. gondii infection, chronic *T. gondii* infection, and uninfected control groups, with 10 samples per group **(Line142~145)**.

Comment 6:

(Figure 3) Which MAG sets, or what metagenomic data, were actually used to describe the mouse toxoplasma study results? How was this model set up? Context is needed.

Response: We thank the reviewer for this comment. We agree that the data and analytical setup for Fig. 3 were not sufficiently clear in the previous version. We have revised the Methods, Results legend to clarify that the mouse *T. gondii* infection results were based on 30 mouse gut shotgun metagenomic samples from NCBI BioProject PRJNA1419542, including AI, CI, and control groups with 10 samples per group **(Line135~145,Line 299~303)**.

We also clarified that these metagenomic reads were mapped to the curated mouse gut vitamin biosynthesis genome dataset, and that TPM-normalized abundances of vitamin biosynthesis-related KEGG Orthologs were used to calculate pathway-level abundance profiles, alpha-diversity metrics, beta-diversity, and taxonomic contributions **(Line 299~303,Line 190~195)**. These revisions provide the necessary context for the Fig. 3 analyses and clarify how the infection-stage comparisons were performed.

Comment 7:

There is no description of infection stages, so it is difficult to assess importance of microbiota comparisons between these 'groups' (lines 271+). Importantly, what kinds of samples this metagenomic sequencing was extracted from is never described.

Response: We thank the reviewer for this comment. We have revised the Materials and Methods section to clarify the data source, sample type, and infection-stage grouping **(Line135~145)**. These revisions clarify what samples were used and provide the necessary context for the microbiota comparisons.

Comment 8:

What were overall microbiota differences across the infection groups? Does vit K differences alone account for this? If general microbiota richness is also decreased during acute infection, the decrease in vit K might just be a reflection of this. Would suggest showing taxonomic / overall functional microbiota richness across the different groups, then comparing whether vit K pathways specifically differ from these trends.

Response: We thank the reviewer for this important comment. Because the present study focused on genome-resolved microbial vitamin B and vitamin K2 biosynthetic potential, we did not systematically compare vitamin pathway changes with global taxonomic richness or overall functional richness.

To address this concern, we revised the Discussion to avoid overinterpreting vitamin K_2-related or vitamin biosynthesis-related changes as pathway-specific effects alone. We also softened related statements in the Discussion and added a limitation noting that the observed changes in vitamin biosynthesis, including vitamin K_2-related pathways, may partly reflect broader microbiome perturbation during acute infection. We further noted that future studies integrating overall taxonomic profiling, global functional profiling, and metabolite measurements will be needed to determine whether vitamin biosynthesis pathways differ from general microbiome trends during *T. gondii* infection **(Line374~381)**.

Comment 9:

How exactly was taxonomic profiling of vit K synthesis genes conducted compared in Figure 3D?

Response: We thank the reviewer for this comment. We have revised the Methods section to clarify how taxonomic profiling of vitamin biosynthesis genes was performed for Fig. 3D **(Line 190~195)**. Specifically, each vitamin biosynthesis-related gene was assigned the taxonomy of its source MAG, which had been classified using GTDB-Tk. TPM-normalized gene abundances were then summed according to source-MAG taxonomy to estimate phylum- and genus-level contributions to vitamin biosynthesis. This clarification explains how the taxonomic profiles shown in Fig. 3D were generated.

Comment 10:

(lines 283 - 289) Why was niacin singled out here, as other vit K pathways also appear to display significantly higher abundances? Relatedly, Figure 3D does not appear to support the associated statement in the results.

Response: We thank the reviewer for this helpful comment. We agree that the previous version did not sufficiently explain why niacin was highlighted and that the Results description associated with Fig. 3D required clarification. We have revised the Results section to clarify that niacin was not singled out because it was the only vitamin pathway showing significant changes. Other vitamin biosynthesis pathways, including vitamin K_2-related pathways, also showed infection-associated differences. Niacin was further emphasized because of its direct connection to NAD^+ and $NADP^+$ metabolism, redox homeostasis, energy metabolism, and immune responses during infection **(Line 313~316)**.

We also rechecked the consistency between Fig. 3D and the corresponding Results text. We also revised the related Results text, figure legend, and supplementary information to ensure that the figures accurately reflect the reported findings.

Comment 11:

MAG generation from samples collected in current study are insufficiently described. What programs were used to do this?

Response: We thank the reviewer for this comment. We apologize for the unclear description. No MAGs were generated from newly collected samples in the present study. All MAGs were obtained from publicly available datasets, including the CMMG dataset and NCBI BioProject PRJNA1419542. We have revised the Materials and Methods section to clearly describe the MAG sources **(Line 135~145)**.

Comment 12:

Were toxoplasma-associated metagenomic data also from previously deposited info, or generated in this study? There is 1 reference (#18) from which most MAGs were built from, but this appears to be just to set up a database. There is also another study (possibly?) from which toxoplasma-specific datasets were derived from, but no reference is given. However, public repository information is given here. If from a previous study, some additional description of the study design would also be helpful for context.

Response: We thank the reviewer for this helpful comment. We apologize that the previous version did not clearly distinguish the sources and purposes of the datasets used in this study. We have revised the Materials and Methods and Data availability sections to clarify that no new sequencing data were generated in the present study. All MAGs and metagenomic data were obtained from previously deposited public resources **(Line 135~145)**.

Comment 13:

A supplemental table specifying the MAGs generated as part of this study, as well as which specific previously deposited MAGs were actually used in the study and their associated metadata (i.e., taxonomic classification, specific accession number, MAG QC info, etc), would be helpful.

Response: We thank the reviewer for this helpful suggestion. We clarify that no

MAGs were newly generated in the present study; all MAGs were downloaded from previously deposited public resources. We have revised the Methods section to clearly describe the MAG sources and accession information, including the CMMG dataset and NCBI BioProject PRJNA1419542 **(Line 135~145).** In addition, the taxonomic classification and genome quality information of the 4,771 MAGs used in this study are provided in the supplementary table.

Comment 14:

Figures seems to contain more analyses (phylogenetic trees, etc) that are never described in Methods.

Response: We thank the reviewer for this helpful comment. We have revised the Methods section to describe how the phylogenetic tree in Fig. 2 was constructed and visualized **(218~220).**

Responses to the comments and suggestions of reviewer #2:

Comment 1:

How does the microbiome change upon Toxoplasma infections alter the mouse physiology and nutrient perspective? Or can the changes help the host resist Toxoplasma infection? Although the authors stated a few scenarios in the discussion, why are they important for the host to adapt to this change? For example, how does the reduction of cobalamin production during acute infection affect the infection from the parasite and/or host sides?

Response: We thank the reviewer for this insightful comment. We have revised the Discussion to clarify how changes in microbial vitamin biosynthetic potential may influence host–microbiome metabolic adaptation and the local nutritional environment during *T. gondii* infection.

In particular, we added a discussion of the observed reduction in microbial cobalamin biosynthetic potential during acute infection. We now explain that this change may reflect disruption of bacterial taxa involved in carbon metabolism,

fermentation, and microbial cross-feeding, with possible implications for host energy metabolism, mucosal immune homeostasis, and the parasite-associated nutritional environment. We also clarified that vitamin concentrations, host physiological responses, and parasite burden were not directly measured in this study; therefore, these interpretations remain hypothesis-generating and require future metabolomic and functional validation.**(Line 374~381)**

Comment 2:

Besides nutrient genes, it would be more interesting to see if other essential nutrient production for Toxoplasma gets changed upon interaction, for example, purines, cholesterol, tryptophan, and arginine, which are essential nutrients for Toxoplasma growth. Reducing the production of these nutrients in the mouse microbiome could limit parasite growth. Therefore, the broad view of these nutrient metabolism genes from the comparison would strengthen the manuscript.

Response: We thank the reviewer for this valuable suggestion. We agree that purine, sterol/cholesterol-related, tryptophan, and arginine metabolism are important nutrient pathways relevant to *T. gondii* growth and host–parasite interactions.

Because the present study was designed to focus specifically on genome-resolved microbial vitamin B and vitamin K_2 biosynthetic potential, particularly niacin/NAD^+-related functions, we did not systematically analyze these broader nutrient metabolism pathways. We have revised the Discussion to acknowledge the importance of these additional nutrient-related pathways and to clarify this as a future research direction**(Line 453~463)**.

Comment 3:

Line 87 has a typo. It should be "linked."

Response: We thank the reviewer for pointing this out. We have corrected the typo in line 87 by changing “inked” to “linked.”**(Line 96)**

We have carefully proofread the manuscript and corrected minor errors and typographical issues throughout the text.

We sincerely hope that the revised manuscript meets your expectations, and we look forward to your editorial decision.

Sincerely yours,

Xiao-Xuan Zhang, BVSc, MVSc, PhD
Qingdao Agricultural University,
Qingdao, Shandong Province 266109,
The People's Republic of China,
Email: zhangxiaoxuan1988@126.com,

Re: Spectrum01564-26R1 (**Genome-resolved analysis reveals disruption of gut microbial vitamin B and K_2 biosynthesis during *Toxoplasma gondii* infection in mice**)

Dear Prof. Xiao-Xuan Zhang:

Thank you for the privilege of reviewing your work. Below you will find my comments, instructions from the Spectrum editorial office, and the reviewer comments.

Thank you for your revisions. While the reviewers appreciate the efforts made to address their previous comments, particularly the helpful clarifications regarding specific datasets and the inclusion of necessary caveats in the Discussion. Some data presentations remain ambiguous. Please refer to the detailed comments included in this letter. To resolve this, we ask that you carefully review and edit the Results section and figure legends to ensure the data is presented clearly and accurately.

Please return the manuscript within 60 days; if you cannot complete the modification within this time period, please contact me. If you do not wish to modify the manuscript and prefer to submit it to another journal, notify me immediately so that the manuscript may be formally withdrawn from consideration by Spectrum.

Revision Guidelines
To submit your modified manuscript, log into the submission site at https://spectrum.msubmit.net/cgi-bin/main.plex. Go to Author Tasks and click the appropriate manuscript title to begin. The information you entered when you first submitted the paper will be displayed; update this as necessary. Note the following requirements:

• Upload point-by-point responses to the issues raised by the reviewers in a file named "Response to Reviewers," NOT in your cover letter.
• Upload a compare copy of the manuscript (without figures) as a "Marked-Up Manuscript" file.
• Upload a clean .DOC/.DOCX version of the revised manuscript and remove the previous version.
• Each figure must be uploaded as a separate, editable, high-resolution file (TIFF or EPS preferred), and any multipanel figures must be assembled into one file.
• Any supplemental material intended for posting by ASM should be uploaded with their legends separate from the main manuscript. You can combine all supplemental material into one file (preferred) or split it into a maximum of 10 files with all associated legends included.

For complete guidelines on revision requirements, see our Submission and Review Process webpage. Submission of a paper that does not conform to guidelines may delay acceptance of your manuscript.

Data availability: ASM policy requires that data be available to the public upon online posting of the article, so please verify all links to sequence records, if present, and make sure that each number retrieves the full record of the data. If a new accession number is not linked or a link is broken, provide Spectrum production staff with the correct URL for the record. If the accession numbers for new data are not publicly accessible before the expected online posting of the article, publication may be delayed; please contact production staff (Spectrum@asmusa.org) immediately with the expected release date.

Publication Fees: For information on publication fees and which article types are subject to charges, visit our website. If your manuscript is accepted for publication and any fees apply, you will be contacted separately about payment during the production process; please follow the instructions in that e-mail. Arrangements for payment must be made before your article is published.

ASM Membership: Corresponding authors may join or renew ASM membership to obtain discounts on publication fees. Need to upgrade your membership level? Please contact Customer Service at Service@asmusa.org.

The ASM Journals program strives for constant improvement in our submission and publication process. Please tell us how we can improve your experience by taking this quick Author Survey.

Thank you for submitting your paper to Spectrum.

Sincerely,
Zhicheng Dou
Editor
Microbiology Spectrum

Reviewer #1 (Comments for the Author):

The authors have clarified one of the major previous comments (clarification of datasets used for the analysis and associated

methods). A few comments still remain related to the author responses:
While the authors have clarified where some of their datasets come from, there are still a few points to address.
- CMMG acronym is never defined
- The authors refer to CMMG as containing only mouse MAGs. If so, where are the different host assignments (human, chicken, cats, sheep, Tibetan antelope?) coming from in Figure S4 (lines 281 - 293)? If the authors added more MAGs from other datasets, where did these datasets come from?
- Although the authors mention revising figure legends, Figure 3 legend remains unrevised. For this and other legends, it is recommended to be very specific about what data is being presented (i.e., shannon diversity calculated from vitamin KOs, overall taxonomy, etc).
- Still missing some context for mouse infection study-while the dataset is listed and what kind of data (metagenomic vs MAGs) is clarified, there is still no description of the original study to give context.

Re: Revised Manuscript ID Spectrum01564-26

Dear Editors and Reviewers

On behalf of all authors, we sincerely thank the Editor and reviewers for their careful evaluation of our manuscript (**Manuscript ID: Spectrum01564-26**) and for their constructive comments and suggestions. In response to these comments, we have carefully revised the manuscript throughout. Specifically, we have defined the CMMG acronym at its first occurrence, clarified the source and use of the cross-host comparative data presented in Supplementary Fig. 4, revised the figure legends to more explicitly describe the data and analytical levels shown in each panel, and added further details on the original mouse infection experiment underlying BioProject PRJNA1419542. We have also carefully checked the manuscript for consistency and clarity and revised the relevant text accordingly. Detailed point-by-point responses to each comment are provided below.

Authors' replies to reviewers' comments

Responses to the comments and suggestions of reviewer #1:

Comment 1:

The authors have clarified one of the major previous comments (clarification of datasets used for the analysis and associated methods). A few comments still remain related to the author responses:

While the authors have clarified where some of their datasets come from, there are still a few points to address.

- CMMG acronym is never defined

Response: Thank you for pointing this out. We have now defined CMMG at its first occurrence as the **Comprehensive Mouse Microbiota Genome** (CMMG) catalog, which refers to the mouse gut genome resource reported by **Kieser et al. (2022) (Reference 19)**. The corresponding text in the Introduction and Materials and Methods has been revised accordingly **(Line 122~124,Line 138~140).**

Comment 2:

The authors refer to CMMG as containing only mouse MAGs. If so, where are the different host assignments (human, chicken, cats, sheep, Tibetan antelope?) coming from in Figure S4 (lines 281 - 293)? If the authors added more MAGs from other datasets, where did these datasets come from?

Response: Thank you for pointing out this ambiguity. We apologize that our original description was not sufficiently clear. The 45,697 MAGs used to construct the genome catalog in the present study were exclusively derived from mice. No MAGs from humans, chickens, cats, sheep, or Tibetan antelope were incorporated into the mouse genome catalog or reanalyzed in this study. The cross-host comparison involving these species had already been presented in the supplementary materials of our previous study (**Reference 9: Yu HL, Elsheikha HM, Liang HR, et al. 2026. Blastocystis infection enhances vitamins B and K_2 biosynthesis in the Tibetan antelope (Pantholops hodgsonii) gut microbiota. BMC Genomics 27:40**). In the present study, we generated the corresponding phylum-level distribution for vitamin-producing genomes in mice and incorporated the mouse result into this previously established cross-host comparison. We have revised the **Materials and Methods(Line 185~189)**, **Results(Line 299~302)** to clarify the source of the comparative data and the role of the mouse dataset in this analysis.

Comment 3:

Although the authors mention revising figure legends, Figure 3 legend remains unrevised. For this and other legends, it is recommended to be very specific about what data is being presented (i.e., shannon diversity calculated from vitamin KOs, overall taxonomy, etc).

Response: Thank you for this helpful comment. We agree that the original figure legends did not sufficiently specify the data underlying each analysis. We have therefore revised the legend of Figure 3 to explicitly describe the data used in each panel (**Line 556~570)**.

Comment 4:

Still missing some context for mouse infection study-while the dataset is listed and what kind of data (metagenomic vs MAGs) is clarified, there is still no description of the original study to give context.

Response: Thank you for this helpful comment. We agree that the previous version did not provide sufficient information regarding the original mouse infection experiment underlying the metagenomic dataset. We have therefore expanded the "Study design and datasets" section to provide additional experimental context **(Line 142~152**).

We have carefully proofread the manuscript and corrected minor errors and typographical issues throughout the text.

We sincerely hope that the revised manuscript meets your expectations, and we look forward to your editorial decision.

Sincerely yours,

Xiao-Xuan Zhang, BVSc, MVSc, PhD
Qingdao Agricultural University,
Qingdao, Shandong Province 266109,
The People's Republic of China,
Email: zhangxiaoxuan1988@126.com,

Re: Spectrum01564-26R2 (**Genome-resolved analysis reveals disruption of gut microbial vitamin B and K_2 biosynthesis during *Toxoplasma gondii* infection in mice**)

Dear Prof. Xiao-Xuan Zhang:

Your manuscript has been accepted, and I am forwarding it to the ASM production staff for publication. Your paper will first be checked to make sure all elements meet the technical requirements. ASM staff will contact you if anything needs to be revised before copyediting and production can begin. Otherwise, you will be notified when your proofs are ready to be viewed.

Data Availability: ASM policy requires that data be available to the public upon online posting of the article, so please verify all links to sequence records, if present, and make sure that each number retrieves the full record of the data. If a new accession number is not linked or a link is broken, provide production staff with the correct URL for the record. If the accession numbers for new data are not publicly accessible before the expected online posting of the article, publication may be delayed; please contact ASM production staff immediately with the expected release date.

Publication Fees: For information on publication fees and which article types have charges, please visit our website. We have partnered with Copyright Clearance Center (CCC) to collect author charges. If fees apply to your paper, you will receive a message from no-reply@copyright.com with further instructions. For questions related to paying charges through RightsLink, please contact CCC at ASM_Support@copyright.com or toll free at +1-877-622-5543. CCC makes every attempt to respond to all emails within 24 hours.

ASM Membership: Corresponding authors may join or renew ASM membership to obtain discounts on publication fees. Need to upgrade your membership level? Please contact Customer Service at Service@asmusa.org.

Embargo Policy: A press release may be issued as soon as the manuscript is posted on the Spectrum Latest Articles webpage. The corresponding author will receive an email with the subject line "ASM Journals Author Services Notification" when the article is available online.

Cover Image Submissions: If you would like to submit a potential Cover Image, please email a file and a short legend to spectrum@asmusa.org. Please note that we can only consider images that (i) the authors created or own and (ii) have not been previously published. By submitting, you agree that the image can be used under the same terms as the published article. Image File requirements: TIF/EPS, 7.5 inches wide by 8.25 inches tall (at least 2,250 pixels wide by 2,475 pixels tall), minimum 300 dpi resolution (600 dpi preferred), RGB, and no figure elements, e.g., arrows or panel labels. The legend should be a short description of the image, 1-2 sentences recommended. Please use the ASM Journals Cover Image Sizing Tool (https://journals.asm.org/pb-assets/html/asm-cover-sizing-tool.html) to ensure that your proposed cover image meets our size requirements.

The ASM Journals program strives for constant improvement in our submission and publication process. Please tell us how we can improve your experience by taking this quick Author Survey.

Thank you for submitting your paper to Spectrum.

Sincerely,
Zhicheng Dou
Editor
Microbiology Spectrum
