# Supplementary material for: Genome-resolved analysis reveals disruption of gut microbial vitamin B and K2 biosynthesis during Toxoplasma gondii infection in mice
Source: Microbiol Spectr. 2026 Sep 8;14(10):e01564-26. doi: 10.1128/spectrum.01564-26 (PMC13688803; doi:10.1128/spectrum.01564-26)
Supplement: Supplementary file 2 [file spectrum.01564-26-s0001.docx]

**Supplementary Information**

**Genome-resolved analysis reveals disruption of gut microbial vitamin B and K_2_ biosynthesis during Toxoplasma gondii infection in mice**

**Hai-Long Yu^1,2,3†^, Hany M. Elsheikha^4†^, He Zhang^2†^, Hai-Ping Wang^5^, Rui Liu^5^, He Ma^5^, Jing Jiang^1*^, Chang-You Xia^2*^, Xiao-Xuan Zhang^5*^**

^1^ College of Life Sciences, Changchun Sci-Tech University, Shuangyang, Jilin Province, PR China.

^2^ State Key Laboratory for Animal Disease Control and Prevention, Harbin Veterinary Research Institute, Chinese Academy of Agricultural Sciences, Harbin, China

^3^ College of Veterinary Medicine, Jilin Agricultural University, Changchun, Jilin Province, PR China.

^4^ Faculty of Medicine and Health Sciences, School of Veterinary Medicine and Science, University of Nottingham, Sutton Bonington Campus, Loughborough, United Kingdom.

^5^ College of Veterinary Medicine, Qingdao Agricultural University, Qingdao, Shandong Province, PR China.

**^*^**Correspondence:

E-mail: jiangjingxiaoyao@163.com (J. Jiang); xiachangyou@caas.cn (C.Y. Xia); zhangxiaoxuan1988@126.com (Xiao-Xuan Zhang)

**^†^** These authors contributed equally to this work.

**Supplementary Fig.1:** Microbial biosynthesis pathways of thiamine, cobalamin, and niacin in the mouse gut.

**Supplementary Fig.2:** Biosynthesis pathways of pantothenate, pyridoxine, and riboflavin.

**Supplementary Fig.3:** Biosynthesis pathways of biotin, folate, and menaquinone.

**Supplementary Fig. 4:** Cross-host comparison of taxonomic contributors to vitamin biosynthesis. Pie charts depict phylum-level distribution of microbial genomes encoding *de novo* vitamin biosynthesis in multiple animal hosts.

**Supplementary Fig.5:** Phylum- and genus-level variation in vitamin biosynthesis genes among groups. (A) Phylum-level differences; (B) Genus-level differences.

**Supplemental Materials**

**Supplementary Table S1**: Overview of 4,771 non-redundant MAGs from the mouse gut microbiome.

**Supplementary Table S2**: KEGG-based functional annotation of microbial genes associated with B-vitamin and vitamin K_2_ biosynthesis pathways.

**Supplementary Table S3**: Overview of vitamin biosynthetic potential and taxonomic classification across 2,682 high-quality genomes.

**Supplementary Table S4**: Overview of 1,665 high-quality genomes with the capacity to independently synthesize at least one vitamin.
